# Application of hyaluronic acid/carboxymethyl cellulose membrane for early continence after nerve-sparing robot-assisted radical prostatectomy

**DOI:** 10.1186/s12894-019-0458-4

**Published:** 2019-04-23

**Authors:** Nobuyuki Hinata, Yukari Bando, Koji Chiba, Junya Furukawa, Kenichi Harada, Takeshi Ishimura, Yuzo Nakano, Masato Fujisawa

**Affiliations:** 1 Department of Urology, Kobe University Graduate School of Medicine, 7-5-1 Kusunoki-cho Chuo-ku, Kobe, 650-0017 Japan; 2 Training Center for Advanced Surgery and Endoscopy, Kobe University School of Medicine, Kobe, Japan

**Keywords:** Prostatectomy, Surgical procedures, robotic, Sodium hyaluronate, carboxycellulose

## Abstract

**Background:**

To assess whether application of a hyaluronic acid-carboxymethyl cellulose membrane (HA/CMC) to the prostate bed and neurovascular plate facilitated early return of continence after nerve-sparing robot-assisted radical prostatectomy (RARP).

**Methods:**

The subjects were 183 consecutive patients with organ-confined prostate cancer who underwent unilateral or bilateral nerve-sparing RARP. After vesicourethral anastomosis, HA/CMC was placed to cover Denonvilliers’ fascia (behind the anastomotic suture) and the preserved neurovascular plate. The time until complete continence after RARP and perioperative complications were compared between patients with or without HA/CMC.

**Results:**

HA/CMC was applied in 13/46 patients (28.3%) receiving bilateral nerve-sparing surgery and 40/137 patients (29.2%) receiving unilateral nerve-sparing surgery. After bilateral nerve-sparing RARP, the median time until continence was significantly shorter in patients with HA/CMC than in those without HA/CMC (3.2 vs. 9.3 months, respectively, *p* < 0.01). After unilateral nerve-sparing RARP, the median time until continence was also significantly shorter in patients with HA/CMC than in those without HA/CMC (3.2 vs. 12.0 months, respectively, p < 0.01). Multivariate Cox proportional hazards regression analysis showed that an age < 70 years (hazard ratio [HR]: 1.74, 95% confidence interval [CI]: 1.12–2.80), institutional caseload > 200, (HR: 1.64, 95%CI: 1.10–2.47), and use of HA/CMC (HR: 1.84, 95%CI: 1.22–2.76) were independent predictors of early postoperative continence. Complication rates, including urinary leakage, did not differ significantly between patients with or without HA/CMC.

**Conclusion:**

Application of HA/CMC to the prostate bed and neurovascular plate resulted in significantly faster postoperative return of continence after both unilateral and bilateral nerve-sparing RARP.

## Background

In men who undergo radical prostatectomy, urinary incontinence is one of the most unwanted postoperative complications [1], and it is well known to have multiple causes [2]. Some studies have shown that fibrosis of periurethral tissue is a risk factor for postoperative incontinence after radical prostatectomy [3, 4].

Elasticity is critical to the function of organs that undergo repeated expansion and contraction [5], and loss of elasticity due to elastic fiber damage is a major contributor to degeneration of connective tissue [6]. Hyaluronic acid (HA) is an important component of the extracellular matrix that coexists with elastic fibers and cooperates functionally with these fibers in various organs/tissues of the human body [7, 8]. HA can increase the retention of water around dehydrated elastic fibers, and hydration is enhanced by self-aggregation of HA [9], leading to further accumulation of water near elastic fibers. This improves elastic fiber recoil by increasing the availability of water molecules for hydrophobic groups in the elastin polypeptide chains. These chains become more disordered with increased hydration, allowing them to store more elastic potential energy during distention [10, 11]. We previously demonstrated the coexistence of elastic fibers and HA in the urethral submucosa and smooth muscle sphincter in male human cadavers, and we also reported that HA is distributed throughout the periurethral nerves [12].

Application of a hyaluronic acid/carboxycellulose membrane (HA/CMC) has been used to prevent postoperative visceral adhesions resulting from inflammation and fibrosis [13]. Elastic fibers and HA coexist in the smooth and striated urethral sphincter muscles, which could be a target for prevention and treatment of urethral sphincter failure. Focusing on the efficacy of HA/CMC for preventing postoperative fibrosis and scarring, we hypothesized that application of HA/CMC could improve postoperative continence after radical prostatectomy. Accordingly, the aim of the present study was to investigate whether intraoperative application of HA/CMC to the prostatic bed could maintain the elasticity of periurethral connective tissue and preserve nerves after surgical invasion, thus facilitating postoperative return of continence after radical prostatectomy.

## Methods

**Fig. 1:**
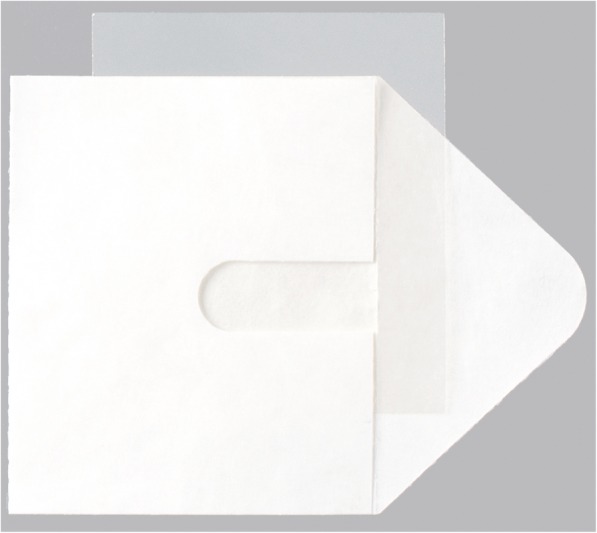
A hyaluronic acid/carboxycellulose membrane (HA/CMC) used in the present study. The length of the membrane was 6.35 cm and the width was 7.35 cm

Use of an HA/CMC adhesion barrier for prevention of adhesions during abdominopelvic surgery is approved by the US Food and Drug Administration. After receiving approval from our institutional review board (approval No.170108), we conducted a retrospective study by reviewing the Kobe University Hospital database between October 2012 and June 2015. The study population was a cohort of 183 consecutive patients with a diagnosis of organ-confined prostate cancer (clinical T1c-2N0M0) who underwent robot-assisted radical prostatectomy (RARP) using unilateral or bilateral nerve-sparing techniques. Nerve-sparing surgery was defined as use of intrafascial or interfascial nerve-sparing techniques, as described previously [14]. Seprafilm® (Genzyme Corporation, Cambridge, MA) was used as HA/CMC in the present study (Fig. 1). HA/CMC turns into a gel within 24–48 h after placement and stays in place for up to 7 days. By day 7, HA/CMC is resorbed and it is excreted from the body by day 28 [15]. After the posterior reconstruction (“Rocco” stitch) [16], 6.35 cm × 7.35 cm sized HA/CMC was placed to cover Denonvilliers’ fascia behind the anastomotic suture and to cover the preserved neurovascular plate. Then the vesicourethral anastomosis was performed. After the vesicourethral anastomosis, HA/CMC was placed on the ventral side of the anastomosis. The 183 patients were divided into three groups based on their treatment during the study period, since the first 42 patients underwent RARP without HA/CMC, the subsequent 53 patients underwent RARP with HA/CMC, and the final 42 patients received RARP without HA/CMC.

The four surgeons who carried out RARP in this series had all performed more than 40 RARP procedures before the study period. The nerve-sparing technique was applied if the tumor was not located ipsilaterally in the prostate on MRI. We excluded patients who had a history of urinary retention or preoperative incontinence. Demographic information was collected, including the age, body mass index, Charlson comorbidity index, pretreatment prostate specific antigen (PSA), prostate volume, hospital patient number, and clinical stage at enrollment. Perioperative and oncological outcomes were also recorded, including the console time, estimated blood loss, use of HA/CMC, perioperative complications categorized by the Clavien-Dindo classification, final pathologic diagnosis, and biochemical recurrence.

The urethral catheter was routinely removed on postoperative day 7. Continence was defined as freedom from pads and the date when each patient achieved pad-free status was determined by interview. Expanded Prostate Cancer Index Composite (EPIC) questionnaires were mailed to the patients every 3 months postoperatively. Biochemical recurrence was defined as a serum PSA level > 0.2 ng/ml on two separate occasions with an interval of at least 6 weeks.

### Statistical analysis

The clinical and pathological descriptive data are presented as percentages, while continuous variables are presented as the median and interquartile range (IQR). Comparison of categorical valuables was performed by the unpaired t-test or Pearson’s chi-square test, as appropriate. The Kaplan-Meier method was used to estimate the continence rate and curves were compared by employing the log-rank test. Cox proportional hazards regression analysis was performed to assess the prognostic significance of target covariates. All statistical analyses were done with JMP software (version 13.0; SAS Institute, Cary, NC, USA), and *P* < 0.05 was taken to indicate statistical significance.

## Results

Among the 183 consecutive patients who underwent nerve-sparing RARP, none of them were excluded due to a history of urinary retention or incontinence. Consequently, all 183 patients were assessed in this study, including 46 patients who underwent bilateral nerve-sparing surgery and 137 patients who received unilateral nerve-sparing surgery. HA/CMC was used in 13/46 patients (28.3%) undergoing bilateral nerve-sparing RARP and 40/137 patients (29.2%) receiving unilateral nerve-sparing RARP.

The demographic and clinical characteristics of the bilateral and unilateral nerve-sparing RARP groups are shown in Table 1. In both groups, there were no statistically significant differences with respect to age, pretreatment PSA, prostate volume, clinical stage, Gleason score at prostate biopsy, Charlson comorbidity index, institutional caseload at surgery, and follow-up period between the patients with or without HA/CMC. Table 2 summarizes the perioperative, histological, and oncological outcomes. In both the bilateral nerve-sparing and unilateral nerve-sparing RARP groups, the operating time and complication rate did not differ significantly between patients with and without HA/CMC. There was also no significant difference of HA/CMC use between the unilateral and bilateral nerve-sparing RARP groups. Moreover, the final pathological and oncological outcomes showed no significant differences between patients with and without HA/CMC in both groups.

**Table 1:** Demographic and clinical characteristics of the two groups

	Bilateral nerve-sparing RARP (*n* = 46)			Unilateral nerve-sparing RARP (*n* = 137)		
	With HA/CMC (*n* = 13)	Without HA/CMC (*n* = 33)	*P* value	With HA/CMC (*n* = 40)	Without HA/CMC (*n* = 97)	*P* value
Age	64.0 (54.1–67.2)	64.9 (59.5–71.3)	0.18	66.4 (62.5–70.7)	66.0 (63.0–71.0)	0.99
BMI	23.5 (21.3–24.4)	23.1 (21.5–25.5)	0.55	23.6 (21.5–25.0)	23.4 (21.6–25.1)	0.14
Pretreatment PSA	7.7 (6.0–9.2)	7.2 (5.5–11.5)	0.87	7.6 (5.2–10.1)	7.2 (5.3–9.6)	0.63
Prostate volume (cm^2^)	24.5 (14–42.8)	28.0 (20.0–45.5)	0.26	28.0 (20.0–32.3)	25.0 (20.0–34.8)	0.82
Clinical stage			0.74			0.65
cT1c	6 (46.2)	16 (48.5)		8 (20.0)	18 (18.6)	
≥cT2	7 (53.8)	17 (51.5)		32 (80.0)	79 (81.4)	
Gleason sum	6 (6–6.5)	7 (6–7)	0.32	7 (7–8)	7 (6.5–7)	0.13
Charlson comorbidity index, n (%)			0.68			0.43
0	3 (23.1)	9 (27.3)		11 (27.5)	32 (33.0)	
≥1	10 (76.9)	24 (72.7)		29 (72.5)	65 (67.0)	
Institutional caseload> 200, n (%)	10 (76.9)	16 (48.5)	0.08	30 (75.0)	57 (57.7)	0.06

**Table 2:** Perioperative, pathological, and oncological outcomes

	Bilateral nerve-sparing RARP (*n* = 46)			Unilateral nerve-sparing RARP (*n* = 137)		
	With HA/CMC (*n* = 13)	Without HA/CMC (*n* = 33)	*P* value	With HA/CMC (*n* = 40)	Without HA/CMC (*n* = 97)	*P* value
Follow-up period, months: median (IQR)	20.7 (16.3–27.0)	20.1 (14.9–29.4)	0.83	21.6 (16.7–27.0)	20.3 (13.3–28.5)	0.79
Operating time, min: median (IQR)	207 (178–223)	215 (189–249)	0.10	210 (176–234)	233 (203–275)	0.09
Estimated blood loss, mL: median (IQR)	10 (2.5–125)	20 (5–125)	0.09	19 (0–88)	70 (0–105)	0.07
Complications ≥ Clavien G3, n (%)	1 (7.7)	3 (9.1)	0.32	1 (2.5)	2 (2.1)	0.87
Urine leakage at cystography, n (%)	1 (7.7)	3 (9.1)	0.81	4 (10.0)	10 (10.3)	0.96
pT, n (%)			0.81			0.38
pT2	12 (92.3)	28 (84.8)		29 (72.5)	77 (79.4)	
≥ pT3	1 (7.7)	5 (15.2)		11 (27.5)	20 (20.6)	
Gleason sum, n (%)			0.48			0.50
6	4 (30.8)	7 (21.2)		1 (2.5)	3 (3.1)	
7	7 (53.8)	23 (30.4)		31 (77.5)	85 (87.6)	
≥8	2 (15.4)	3 (9.1)		8 (20.0)	9 (9.3)	
Positive surgical margin, n (%)	2 (15.4)	3 (9.1)	0.54	5 (12.5)	15 (15.5)	0.37
Biochemical failure, n (%)	0 (0)	0 (0)	–	2 (5.0)	6 (6.2)	0.79

**Fig. 2:**
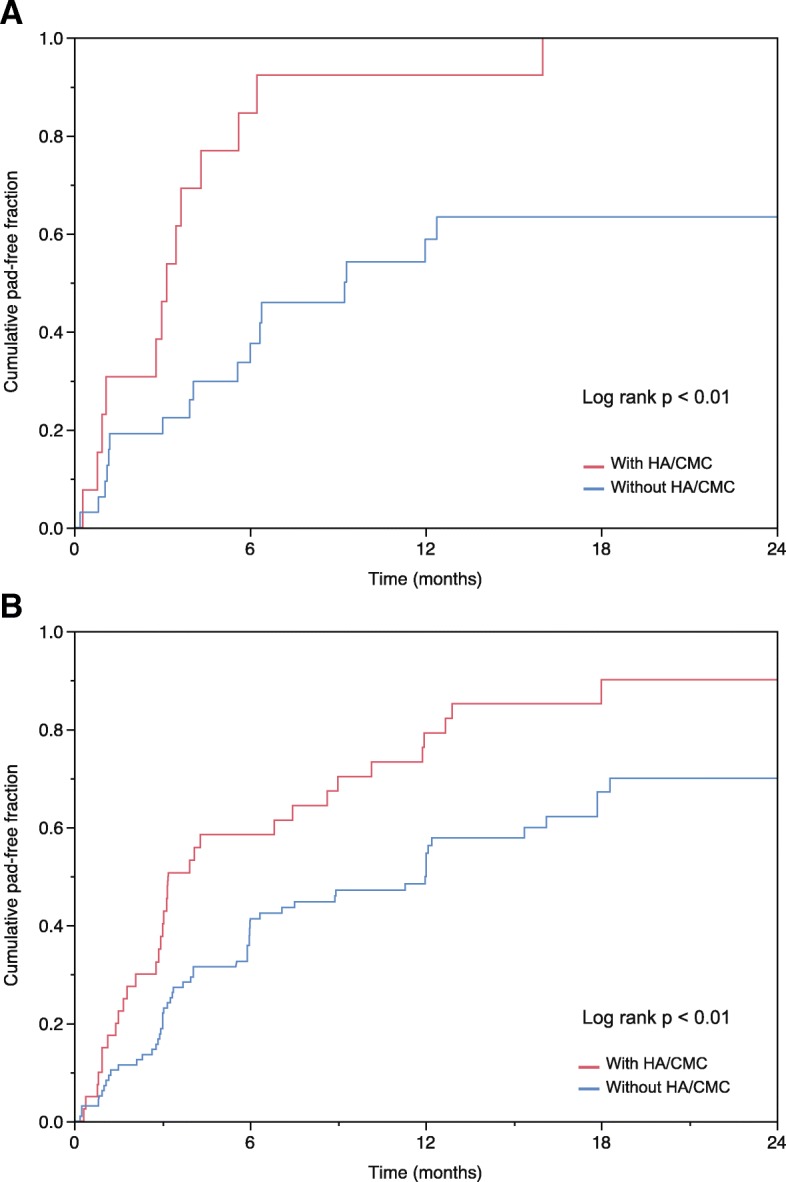
Pad-free status of patients who underwent bilateral nerve-sparing RARP (*n* = 46) (**a**) and unilateral nerve-sparing RARP (*n* = 137) (**b**)

Among the 46 patients who received bilateral nerve-sparing RARP, the median time to achieve continence was significantly shorter in patients with HA/CMC than in those without HA/CMC (3.2 months vs. 9.3 months, respectively, *p* < 0.01) (Fig. 2a). Among the 137 patients who had unilateral nerve-sparing RARP, the median time to continence was also significantly shorter in patients with HA/CMC than in those without HA/CMC (3.2 months vs. 12.0 months, respectively, *p* < 0.01) (Fig. 2b).

Potential predictors of the time until complete postoperative continence are evaluated in Table 3. Univariate analysis showed that the age, institutional caseload, and use of HA/CMC were the predictors of achieving pad-free status. Multivariate Cox analysis indicated that an age < 70 years (hazard ratio [HR]: 1.74, 95% confidence interval [CI]: 1.12–2.80), institutional caseload > 200, (HR: 1.64, 95%CI: 1.10–2.47), and use of HA/CMC (HR: 1.84, 95%CI: 1.22–2.76) were independent predictors of the early return of continence.

**Table 3:** Univariate and multivariate Cox regression analysis of factors influencing achievement of pad-free status

	Univariate analysis			Multivariate analysis		
	HR	95% CI	*P* value	HR	95%CI	*P* value
Age ≤ 70 years	1.67	1.08–2.68	0.02	1.74	1.12–2.80	0.01
BMI ≤24	1.11	0.76–1.66	0.61			
Nerve-sparing (bilateral vs. unilateral)	1.24	0.80–1.87	0.33			
Institutional caseload > 200	1.86	1.28–2.71	<0.01	1.64	1.10–2.47	0.02
Use of HA/CMC	2.26	1.54–3.30	<0.01	1.84	1.22–2.76	<0.01

## Discussion

We investigated the influence of HA/CMC on early return of continence after nerve-sparing RARP and demonstrated that use of HA/CMC during nerve-sparing RARP significantly shortened the duration of postoperative incontinence. This difference was not only seen in patients undergoing bilateral nerve-sparing surgery, but also in those having unilateral nerve-sparing surgery. Multivariate analysis confirmed that use of HA/CMC was an independent predictor of postoperative recovery of continence.

Periurethral fibrosis and scarring have been reported as factors that predict postoperative incontinence after radical prostatectomy [3, 4]. Technical factors such as the amount of postoperative oozing or a prolonged operating time might contribute to fibrotic change. These factors can be improved by increasing the surgeon’s or institution’s experience, but complete prevention of inflammation or fibrosis after surgical invasion is theoretically impossible, and no method of preventing fibrosis or scarring after radical prostatectomy has been reported to our knowledge.

One of the main causes of postoperative incontinence after radical prostatectomy could be periurethral scarring, which can lead to chronic compression and ischemia of the periurethral tissues as well as sphincteric nerves. HA is a major component of the extracellular matrix and it plays an important role in early wound healing together with fibrin [17]. HA stimulates the production of interleukin-1 (IL-1) and IL-1 promotes fibroblast proliferation and collagenase production [18]. HA is also involved in regulating leukocyte motility, adhesion, and phagocytosis, and it acts to suppress scar formation caused by infiltration of inflammatory cells into damaged tissues [19]. HA/CMC is a surgical material with a 66.6% content of HA that is widely been used to prevent visceral adhesion by inhibiting postoperative local inflammation and fibrosis [20]. The improvement of early postoperative continence after RARP observed in the present study could have been due to suppression of periurethral scarring by intraoperative application of HA/CMC. Because HA/CMC is available worldwide, easy to use, and low cost, it seems to be a reasonable option for prevention of scarring of the urethral sphincter and periurethral tissue after radical prostatectomy.

HA also has an important role in maintaining tissue elasticity. HA increases hydration of the extracellular matrix and traps water molecules around elastic fibers, thus improving their function. Elastic fibers are a major insoluble component of the extracellular matrix that permit deformation and passive recoil of organs or tissues [9, 10]. We previously demonstrated co-localization of elastic fibers and HA in the submucosa and smooth muscle of the membranous urethra [12]. Surgical invasion can reduce tissue elasticity derived from water captured by a matrix of HA and elastic fibers, so the elasticity of periurethral tissue could be impaired after radical prostatectomy. Loss of elasticity was reported to lead to the degeneration of connective tissue [6]. In the present study, faster recovery of continence after RARP was confirmed in patients with HA/CMC than in those without it. Although direct measurement of the urethral elasticity is impossible, this difference might have been related to maintenance of urethral elasticity by application of HA/CMC.

**Fig. 3:**
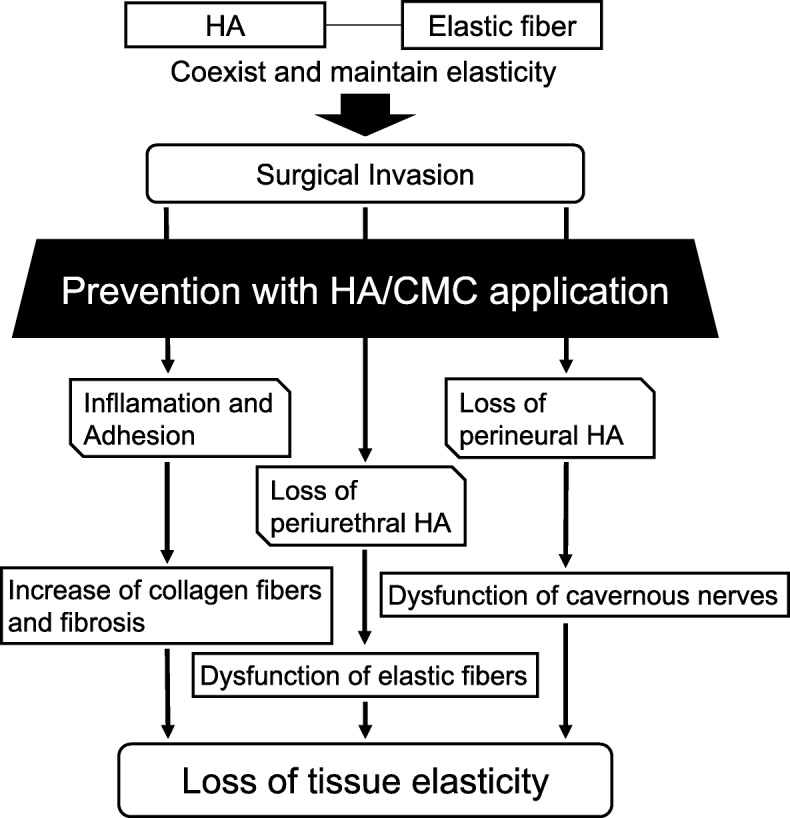
Mechanism by which application of HA/CMC may prevent three possible causes of the postoperative loss of tissue elasticity due to surgical invasion

HA can also prevent cicatrization of peripheral nerves and promote nerve regeneration. For example, rats showed faster functional recovery after sciatic nerve dissection when HA was applied to the dissected nerve [21]. Furthermore, HA creates a hydrated open lattice in the extracellular matrix that facilitates migration of regenerating axons [22]. We previously reported that peripheral nerves running through the hiatus between the bilateral levator ani slings were positive for HA in human male cadavers [12]. An association between sparing the cavernous nerves and early postoperative return of urinary continence has already been described [23]. Repeated injection of HA into nerve conduits was reported to enhance regeneration of sciatic nerve defects in rats [22]. In another study, HA reduced perineural adhesions at 4 weeks and 12 weeks after neurorrhaphy, and also enhanced peripheral nerve regeneration at 12 weeks postoperatively [21]. Furthermore, HA/CMC has been reported to reduce extraneural adhesions and promote nerve regeneration at 3 months after sciatic nerve repair in rabbits [24]. In the present study, early return of continence after application of HA/CMC to the nerve-sparing side may have been related to enhanced regeneration of axons in the cavernous nerves. Figure 3 outlines how application of HA/CMC could prevent three potential causes of postoperative loss of tissue elasticity due to surgical invasion.

A retrospective study showed that wrapping HA/CMC around the suture line led to a higher frequency of sequelae associated with intestinal anastomotic leakage after abdominopelvic surgery [25]. However, covering the prostate bed with HA/CMC did not increase including postoperative urine leakage or other complications in the present study, even though RP involves vesicourethral anastomosis. Joung et al. reported earlier recovery of erectile function when the neurovascular bundle was coated with HA/CMC gel during RARP than in the control group [26]. They also reported that complication rates did not differ between the two groups, a similar finding to the results of the present study. It is possible that precise suturing with robotic assistance [27] could contribute to reducing urine leakage and prevent migration of dissolved HA/CMC after radical prostatectomy. Excellent hemostasis of the prostatic bed achieved with RARP could also have enabled safe application of HA/CMC.

Several limitations of this study should be considered. First, the retrospective design and small number of patients limit the conclusions that can be drawn. Second, reasons of the rather low continence outcomes of the non-HA/CMC groups in the present study might be ascribed to the strict continence definition used and the fact that not all the four surgeons were experts. Third, the treatment protocol was not randomized, so results might have been influenced by the learning curve effect. Thompson et al. reported that RARP had a long learning curve with inferior early results that progressively transform to superior functional and oncological outcomes [28]. Although patients receiving HA/CMC were assigned according to institutional policy, the caseload was still an independent predictor of postoperative continence by Cox proportional hazards analysis in the present study. However, use of HA/CMC was also an independent predictor of the earlier achievement of pad-free status by Cox analysis. Thus, intraoperative application of HA/CMC to the prostate bed during nerve-sparing RARP improved postoperative continence.

## Conclusions

This study demonstrated that application of HA/CMC to the prostate bed and neurovascular plate significantly shortened the duration of postoperative incontinence after both unilateral and bilateral nerve-sparing RARP. Further researches on this working mechanism and a prospective randomized controlled trial of HA/CMC in patients undergoing RARP seems to be warranted.

## Abbreviations

HA: hyaluronic acid
HA/CMC: hyaluronic acid-carboxymethyl cellulose membrane
RARP: robot-assisted radical prostatectomy
